# Analysis of rare variations reveals roles of amino acid residues in the N-terminal extracellular domain of nicotinic acetylcholine receptor (nAChR) alpha6 subunit in the functional expression of human alpha6*-nAChRs

**DOI:** 10.1186/1756-6606-7-35

**Published:** 2014-05-02

**Authors:** Bhagirathi Dash, Ming D Li

**Affiliations:** 1 Department of Psychiatry and Neurobehavioral Sciences, School of Medicine, University of Virginia, Charlottesville, VA, USA

**Keywords:** Electrophysiology, Ion channels, Nicotinic acetylcholine receptor, Receptor structure-function, Single nucleotide variation

## Abstract

**Background:**

Functional heterologous expression of naturally-expressed and apparently functional mammalian α6*-nicotinic acetylcholine receptors (nAChRs; where ‘*’ indicates presence of additional subunits) has been difficult. Here we wanted to investigate the role of N-terminal domain (NTD) residues of human (h) nAChR α6 subunit in the functional expression of hα6*-nAChRs. To this end, instead of adopting random mutagenesis as a tool, we used 15 NTD rare variations (i.e., Ser43Pro, Asn46Lys, Asp57Asn, Arg87Cys, Asp92Glu, Arg96His, Glu101Lys, Ala112Val, Ser156Arg, Asn171Lys, Ala184Asp, Asp199Tyr, Asn203Thr, Ile226Thr and Ser233Cys) in nAChR hα6 subunit to probe for their effect on the functional expression of hα6*-nAChRs.

**Results:**

N-terminal α-helix (Asp^57^); complementary face/inner β-fold (Arg^87^ or Asp^92^) and principal face/outer β-fold (Ser^156^ or Asn^171^) residues in the hα6 subunit are crucial for functional expression of the hα6*-nAChRs as variations in these residues reduce or abrogate the function of hα6hβ2*-, hα6hβ4- and hα6hβ4hβ3-nAChRs. While variations at residues Ser^43^ or Asn^46^ (both in N-terminal α-helix) in hα6 subunit reduce hα6hβ2*-nAChRs function those at residues Arg^96^ (β2-β3 loop), Asp^199^ (loop F) or Ser^233^ (β10-strand) increase hα6hβ2*-nAChR function. Similarly substitution of NTD α-helix (Asn^46^), loop F (Asp^199^), loop A (Ala^112^), loop B (Ala^184^), or loop C (Ile^226^) residues in hα6 subunit increase the function of hα6hβ4-nAChRs. All other variations in hα6 subunit do not affect the function of hα6hβ2*- and hα6hβ4*-nAChRs. Incorporation of nAChR hβ3 subunits always increase the function of wild-type or variant hα6hβ4-nAChRs except for those of hα6(D57N, S156R, R87C or N171K)hβ4-nAChRs. It appears Asp57Lys, Ser156Arg or Asn171Lys variations in hα6 subunit drive the hα6hβ4hβ3-nAChRs into a nonfunctional state as at spontaneously open hα6(D57N, S156R or N171K)hβ4hβ3^V9’S^-nAChRs (V9’S; transmembrane II 9’ valine-to-serine mutation) agonists act as antagonists. Agonist sensitivity of hα6hβ4- and/or hα6hβ4hβ3-nAChRs is nominally increased due to Arg96His, Ala184Asp, Asp199Tyr or Ser233Cys variation in hα6 subunit.

**Conclusions:**

Hence investigating functional consequences of natural variations in nAChR hα6 subunit we have discovered additional bases for cell surface functional expression of various subtypes of hα6*-nAChRs. Variations (Asp57Asn, Arg87Cys, Asp92Glu, Ser156Arg or Asn171Lys) in hα6 subunit that compromise hα6*-nAChR function are expected to contribute to individual differences in responses to smoked nicotine.

## Background

**Figure 1:**
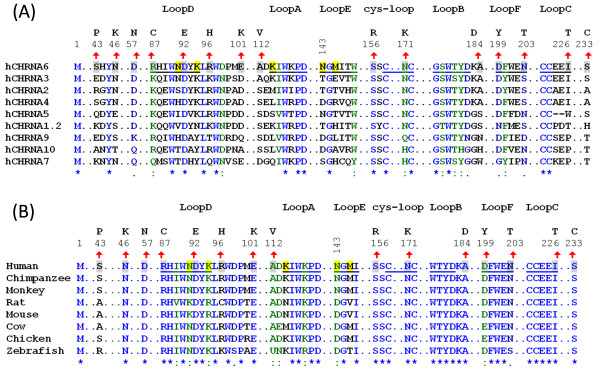
**Localizations of N-terminal variations to primary/secondary structure of nAChR hα6 subunits. (A)** Degree of conservation of variant residues in nAChR hα6 subunit in relation to other human nAChR α subunits: Human nAChR α subunits (α1-α7, α9 and α10) are aligned using ClustalW. Indicated residues in nAChR hα6 subunits undergoing variation are fully (Asp^92^ and Ser^156^), strongly (Arg^87^ and Asn^171^) and weakly (Asp^57^, Asp^199^ and Asn^203^) conserved in human nAChR α subunits. Some of the variations in nAChR hα6 subunit are localized to indicated loop regions: Ala112Val (loop A), Ala184Asp (loop B), Ile226Thr (loop C), Asp92Glu (loop D), Asp199Tyr (loop F) and Asn203Thr (loop F) and Asn171Lys (cysteine-loop). **(B)** Degree of conservation of variant residues in nAChR hα6 subunit in relation to nAChR α6 subunits from other organisms: nAChR α6 protein sequences extracted from (GenBank) NP_067344.2 (Mouse), NP_004189.1 (Human), NP_990695.1 (Chicken), NP_476532.1 (Rat), NP_001029266.1 (Chimpanzee), XP_001099152.1 (Monkey), XP_584902.3 (Cow) and NP_001036149.1 (Zebrafish) are aligned by using ClustalW. For both **(A)** and **(B)**; numbering begins at translation start methionine of nAChR hα6 subunit and is shown in the regions of interest. However, only segments of the alignment are presented to identify WT nAChR hα6 subunit AA residues (shaded, upward arrow mark and numbers above them) and their corresponding variations (noted above the numberings). Symbols below sequences indicate fully (*), strongly (:) or weakly (.) conserved residues: hα6 subunit AA residues at positions 87 (Arg), 92 (Asp), 156 (Ser) and 171 (Asn) are conserved in both human nAChR α subunits and nAChR α6 subunits of other organisms. Also shown (shaded) are the nAChR hα6 subunit residues including the loop E residue N143 that alone or in combination Met145 influences the function of hα6*-nAChRs [26].

**Figure 2:**
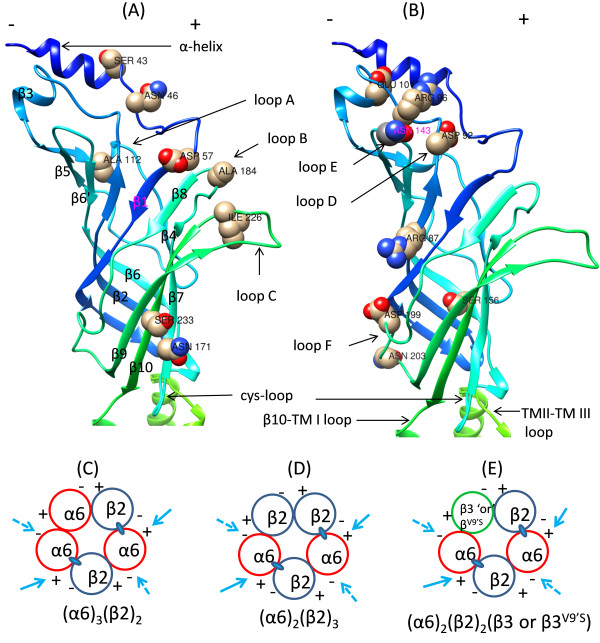
**Localizations of AA variations to secondary structures and interfaces of nAChR hα6 subunit. [(A) and (B)]** AA residues undergoing variation are identified in a 3D model of the nAChR hα6 subunit: A 3D homology model of the nAChR hα6 subunit was generated based on the crystal structure of *Torped*o muscle nAChR α subunit (PDB code: 2BG9:A). Hence structural features are approximate and may deviate from what seen with α subunit of *Torped*o muscle nAChR. β strands constitute inner β-sheet (strand β1-β3, β5, β6 and β8) and outer β-sheet (strand β4, β7, β9 and β10); and are connect to each other via loops. These loops constitute positive (+) (loops A, B and C) and negative face (loops D, E and F) of α6 subunit and contribute to subunit assembly, ligand binding, and formation of ligand binding pocket and/or coupling agonist binding to channel gating. Cysteine loop and other loop residues undergone variations are also identified. **[(C)-(E)]** Interfaces contributed by α6 subunit to formation of α6*-nAChR: Adhering to the canonical rule of pentamer formation, α6β2-nAChR would be formed out of three α6 and two β2 subunits [i.e., (i) (α6)_3_(β2)_2_-nAChR] or two α6 and three β2 subunits [i.e., (ii) (α6)_2_(β2)_3_-nAChR]. In the event β3 or gain-of-function β3 (i.e., β3^V9’S^) subunits to be integrated into α6β2*-nAChR complexes these subunits would take the position of 3^rd^ α6 subunit in the 1^st^ (i) configuration or 3^rd^ β2 subunit in the 2^nd^ (ii) configuration [i.e., (iii) (α6)_2_(β2)_2_(β3or β3^V9’S^)-nAChR]. Similarly, β4 subunit would substitute β2 subunit for formation human α6β4-[i.e., (α6)_3_(β4)_2_ and (α6)_2_(β4)_3_]-nAChR. For formation of (α6)_2_(β4)_2_β3- or (α6)_2_(β4)_2_β3^V9’S^-nAChR β3 or β3^V9’S^ subunits would substitute one α6 subunit in (α6)_3_(β4)_2_ configuration or one β4 subunit in (α6)_2_(β4)_3_ configuration. Two presumed agonist (ACh or nicotine and others) binding sites in the interface of α6(+) and β2(−) subunits are identified as ovals. Variations in the structural loops in the (−)-ve face (loop D, E and F) and (+)-ve face (loop A, B and C) of the hα6 subunit are expected to affect the function of hα6*-nAChRs involving interfaces identified by arrow marks.

Mammalian neuronal nicotinic acetylcholine receptors (nAChRs) are formed out of six α subunits (α2-α7) and three β subunits (β2-β4) [1] where in five subunits either α (e.g., α7, α9α10; etc.) or α plus β come together (e.g., α4β2, α4β2α5, α6β2, α6β2β3, α6β4, α7β2; etc.) to form a ligand gated ion channel [1]. Each nAChR subunit has a large extracellular N-terminal domain (E1 or NTD), followed by four transmembrane domains (TM I, II, III and IV) and a small C-terminal domain (CTD). TM domains are connected to each other via loops: a small intracellular loop (C1) connects TM I to TM II, an extracellular loop (E2) connects TM II to TM III and a large intracellular loop (C2) connects TM III to TM IV. The NTD of a human nAChR subunit is presumed to contain an inner β-sheet (composed of strands β1-β3, β5, β6 and β8) and outer β-sheet (composed of strands β4, β7, β9 and β10) like those typically seen in the crystal structure of *Torpedo* muscle nAChR or other eukaryotic and prokaryotic ACh binding protein subunits [2-5]. The β-strands connect to each other via loops: they are known as loops A, B and C in the principal or positive (+) face and loops D, E and F in the complementary or negative (−) face (Figures 1 and 2). These loops and additional residues in the NTD of participating α and β subunits form the ligand-binding site for nAChRs. The β6-β7 loop has a pair of disulfide-bonded cysteines separated by 13 residues that form the cysteine-loop (i.e., cys-loop) motif and it is essential for nAChR assembly and channel gating [6,7].

Mammalian α6*-nAChRs (where ‘*’ indicates presence of additional subunits) naturally exist as combinations of α6 with β2 or β4 alone or with addition of β3 subunits [8-17]. α6β2*-nAChRs are proven to be physiologically important: they play a role in the modulation of dopamine release, reward and reinforcement behavior, and psychiatric diseases such as schizophrenia, Parkinson’s disease; etc. [11-13,15,18-21]. The functional role of α6β4*-nAChRs is poorly understood. Therefore *in vitro* functional expression and characterization of these nAChRs are highly desirable for development of α6*-nAChR specific ligands to treat diseases associated with them including ND [22-24]. However, functional heterologous expression of mouse or human (h) α6*-nAChRs has been difficult. There is not emergence of functional α6β2*-nAChRs although poorly functioning α6β4*-nAChRs could be obtained in some heterologous expression systems [25-31]. In recent years improved success in functional expression of α6*-nAChRs are achieved by employing mutant and/or chimeric nAChR subunits [25-28,32-34].

Some studies suggest that β3 subunits may facilitate α6*-nAChR trafficking to the cell membrane [35]. However, others indicate that coexpression of wild-type (WT) nAChR β3 subunits has a dominant-negative effect on function of α6*-nAChRs [25,26,28]. The dominant negative effect of nAChR β3 subunits could be overcome by coexpression with mutant β3^V9’S^ subunits [i.e., valine(V)-to-serine(S) mutation at 9’ position in the putative second transmembrane domain] [25,26]. Upon further investigation it has been shown that amino acid (AA) residues in the NTD of α6 subunits influence the effects of the WT or mutant β3 subunits (i.e., β3^V9’S^) and are involved in gain-of-function effects of mutant β3^V9’S^ subunits on α6*-nAChR function [26]. These findings also suggest that co-assembly of nAChR β3 subunits with α6 plus β2 or β4 subunits to form functional nAChR is determined to some degree by the α6 subunit N-terminal, extracellular region. This is because improved success in functional expression of α6*-nAChRs is obtained when chimeric mouse-human α6 subunits (where the N-terminal domain of mouse α6 subunit is fused to the rest of human α6 subunits) are employed instead of WT hα6 subunit. This has led to the discovery of Asn143 in the putative loop E region of the NTD of hα6 subunit as a key determinant for the heterologous functional expression of α6*-nAChRs [26-28].

Therefore it appears that there probably exist additional residues in the NTD of hα6 subunit some of which will be of crucial importance and mutations in them will not be tolerated, and others which could be target for improved functional expression of α6*-nAChRs. In the absence of these information (and not to use random mutagenesis as a tool towards this end) we reasoned to use naturally occurring rare variations in the NTD of hα6 subunit as probes to uncover N-terminal molecular bases important for functional expression of hα6hβ2*- and hα6hβ4*-nAChRs. To this end we evaluated the effect of 15 naturally occurring rare missense mutations, that occur in various regions of the N-terminal extracellular domain of hα6 subunit [N-terminal α-helix: Ser43Pro, Asn46Lys and Asp57Asn; complementary face/inner β-fold: Arg87Cys (β2-strand); Asp92Glu, Arg96His and Glu101Lys (loop D/β2-β3 loop); Ser156Arg (β6-strand); and Asp199Tyr and Asn203Thr (loop F)] and principal face/outer β-fold: Ala112Val (loop A), Asn171Lys (cysteine loop, potential site for N-glycosylation), Ala184Asp (loop B), Ile226Thr (loop C) and Ser233Cys (β10-strand); see Figures 1 and 2; Table 1], on the functional expression of hα6*-nAChRs. On occasion we took aid of gain-of-function hβ3 subunits (i.e., hβ3^V9’S^ = hβ3^V273S^) (to increase receptor sensitivity) and a codon optimized nAChR hβ2 subunit (to increase receptor expression efficiency) to provide additional insight into the effect of these natural variations on the functional expression of hα6*-nAChRs. Our results indicate that some of the rare variations abrogate, decrease, increase or do not affect the functional expression of hα6hβ2*- and hα6hβ4*-nAChRs. There also appear to be a subtype specific effect of some of these variations. By undertaking this study, as anticipated, we were able to uncover some of the N-terminal molecular bases in hα6 subunit that could be taken advantage for modulating functional expression of hα6hβ2*-, hα6hβ4- and hα6hβ4hβ3-nAChRs. These results provide foundations for undertaking more specialized and individual mutation specific studies later on.

**Table 1:** Characteristics of single nucleotide variants (SNVs) located in the N-terminal domain of nAChR hα6 subunit

**rs ID #**	**Nucleotide position in chromosome 8**	**All [AA + EA] Allele #**	**Amino acid change**	**Change in electrical charge**	**Conservation in human nAChR α subunits**	**Conservation in α6 subunits of other species**	**NTD location**	
rs140930963	8:42620300	G = 37/A = 10721	S43P		No	-		α-helix
rs80342906	8:42620289	C = 6/G = 10752	N46K		No	N: yes		α-helix
rs149966755	8:42620258	T = 1/C = 10757	D57N	‘-‘ve to zero	D: weak	D: yes		α-helix
unknown	8:42614217	A = 2/G = 10756	R87C	‘+’ve to zero	R: strong	R: yes	Complementary face/inner β-sheet	Strand β2
rs146332801	8:42612169	C = 1/A = 10755	D92E		D: fully	D: yes		β2-β3 loop (loop D)
rs188620180	8:42612158	A/G	R96H		No	-		β2-β3 loop
rs200380236	8:42612144	T = 1/C = 10755	E101K	‘-‘ve to ‘+’ve	No	E: yes		β2-β3 loop/^1^MIR
unknown	8:42611874	C = 1/A = 10757	S156R	neutral to ‘+’ve	S: fully	S: yes		Strand β6
unknown	8:42611747	A = 2/C = 10756	D199Y	‘-’ve to neutral	D: weak	D: weak		β8-β9 loop (loop F)
rs143385261	8:42611734	G = 3/T = 10755	N203T		N: weak	N: weak		β8-β9 loop (loop F)
rs141518931	8:42612110	A = 1/G = 10755	A112V		No	A: yes	Principal face/outer β-sheet	β3-β4 loop (Loop A)
rs79945499	8:42611829	G/C	N171K	neutral to ‘+’ve	N: strong	N: yes		β6-β7 (Cysteine loop)
rs200745568	8:42611791	T = 1/G = 10757	A184D	neutral to ‘-‘ve	No	A: yes		β7-β8 loop (loop B)
rs199987912	8:42611665	G = 2/A = 10756	I226T		No	I: yes		β9-β10 loop (loop C)
unknown	8:42611644	C = 1/G = 10757	S233C		No	S: yes		Strand β10

^1^MIR: Main immunogenic region.

Pertinent information about the N-terminal variations in nAChR hα6 subunit such as their reference SNP (rs) identification (ID) number, location in the chromosome 8, combined frequency of nucleotide variations discovered in more than 10000 genomes of African Americans (AA) and European Americans (EA) (Exome Sequencing Project: ESP; https://esp.gs.washington.edu/), change in amino acids, change in electrical charges, conservation in the human nAChR α subunits (Figure 1), conservation in nAChR α6 subunit of other species and putative locations in the secondary structure of the nAChR hα6 subunits (Figure 2) are presented. The WT AAs with the exception of S43 are strongly conserved in nAChR α6 subunits studied from a limited number of other species (Figure 1). Please note that hα6 variations R87C, S156R, D199Y and S233C retrieved from ESP do not have an rs ID number yet. The N-terminal domain of a typical human nAChR subunit is presumed to contain an inner β-sheet (strand β1-β3, β5, β6 and β8) and outer β-sheet (strand β4, β7, β9 and β10) like those of seen in the crystal structure of *Torpedo* muscle nAChR subunits. ‘-‘ indicates lack of indicated information in these positions.

## Results

### Bioinformatic analyses indicate possible functional consequences of rare variations in nAChR α6 subunits

Information retrieved from NCBI dbSNP database, NHLBI Grand Opportunity Exome Sequencing Project (ESP), multiple protein sequence alignment of human nAChR α subunits, multiple protein sequence alignment of nAChR α6 subunits from various model organisms and homology model of nAChR hα6 subunit (Figures 1 and 2) are combined to present an overview of the characteristics of the 15 single nucleotide variations (SNVs) evaluated for their effect on function of hα6*-nAChRs (Table 1). African American and European American populations sampled in the ESP indicate that these nucleotide variations are rare and their combined frequency ranges from 0.001 to 0.3% (Table 1).

Full length protein sequence alignments of human nAChR α subunits indicate that fully conserved (Asp^92^ and Ser^156^), strongly conserved (Arg^87^ and Asn^171^), weakly conserved (Asp^57^, Asp^199^ and Asn^203^), and non-conserved (the rest 8: Ser^43^, Asn^46^, Arg^96^, Glu^101^, Ala^112^, Ala^184^, Ile^226^ and Ser^233^) AAs in the NTD of nAChR hα6 subunit have undergone variations [Figures 1(A)]. Also nAChR hα6 subunit AAs undergoing variation are fully conserved (Asn^46^, Asp^57^, Arg^87^, Asp^92^, Glu^101^, Ser^156^, Asn^171^, Ala^184^, Ile^226^ and Ser^233^), strongly conserved (Ala^112^ and Asp^199^), weakly conserved (Asn^203^) and non-conserved (the rest 2: Ser^43^ and Arg^96^) in an alignment of nAChR α6 subunits from various organisms [Figure 1B]. Together, these results indicate that nAChR hα6 subunit AAs at positions 87 (Arg), 92 (Asp), 156 (Ser), and 171 (Asn) are conserved in both human nAChR α subunits and nAChR α6 subunits from other organisms. Lacking a priori knowledge, we hypothesize that these variations at conserved AAs would have an effect on the structure and/or function of hα6*-nAChRs.

Positions of the WT or variant AAs in nAChR hα6 subunit are mapped to their secondary structural features such as α-helices, β-strands and loops (Figures 1 and 2; Table 1) by sequence alignment and/or comparison to other nAChR subunits and most prominently to that of the muscle nAChR α subunits of *Torpedo marmorata* (PDB code: 2BG9.A) [2]. We hypothesized that some of these variations in nAChR hα6 subunit occurring in the loop A (Ala112Val), loop B (Ala184Asp), loop C (Ile226Thr), loop D/β2-β3 loop (Arg87Cys, Asp92Glu and Arg96His), loop F (Asp199Tyr and Asn203Thr) and cysteine-loop (Asn171Lys) would affect the structure and/or function of hα6*-nAChR as these regions are known to be important in subunit assembly, ligand binding and/or signal transduction of various other subtypes of nAChRs [2,7]. Also change in the electrical properties and other characteristics of the AAs as a result of the variation potentially could impact the intra- and/or inter subunit molecular interactions involving ionic and other types bonds that may affect the structure and/or function of hα6*-nAChRs (Tables 1 and 2).

**Table 2:** Primers used to create single nucleotide variation in nAChR α6 subunit cDNA

**Sl. no**	**Primer**	**Oligonucleotide sequence (5’ → 3’)**
1.	hα6(S43P)	ctcttccacaaactgtttCctcattacaaccagttc
2.	hα6(N46K)	ctgttttctcattacaaGcagttcatcaggcctg
3.	hα6(D57N)	gtggaaaacgtttccAaccctgtcacggtac
4.	hα6(R87C)	gaaaccaatttgtggctgTgtcacatctggaatgatt
5.	hα6(D92E)	cgtcacatctggaatgaGtataaattgcgctggg
6.	hα6(R96H)	ctggaatgattataaattgcActgggatccaatggaatatg
7.	hα6(E101K)	gcgctgggatccaatgAaatatgatggcattgag
8.	hα6(A112V)	gactcttcgcgttcctgTagataagatttggaagc
9.	hα6(S156R)	ccagctatttttaagagGtcctgccctatggatatc
10.	hα6(N171K)	ctttttcccttttgatcatcaaaaGtgttccctaaaatttggttcctgg
11.	hα6(A184D)	cctggacgtatgacaaagAtgaaattgatcttctaatc
12.	hα6(D199Y)	ggatcaaaagtggatatgaatTatttttgggaaaacagtgaatg
13.	hα6(N203T)	gatatgaatgatttttgggaaaCcagtgaatgggaaatcattgatg
14.	hα6(I226T)	caaatacaactgttgtgaagagaCatacacagatataacctattc
15.	hα6(S233C)	gatatacacagatataacctattGtttctacattagaagattgccg

For mutants, the first amino acid (single letter code, numbering begins at the translation start methionine) designates the WT human nAChR hα6 subunit residue that is replaced with the indicated, second amino acid. In the forward primer nucleic acid sequence, capitalization indicates the nucleotide changed from the WT subunit to create the corresponding mutant.

### Current responses are null whether WT nAChR hα6 subunits are expressed in oocytes with WT or codon optimized hβ2 subunits alone or in the additional presence of hβ3 subunits

Functional expressions of human α6β2- and α6β2β3-nAChRs were not achieved in *Xenopus* oocytes whether WT or codon optimized human nAChR β2 subunits were expressed with hα6 subunits alone or in the additional presence of hβ3 subunits.

### Human α6β2β3^V9’S^-nAChRs are functional whether they are expressed using WT or codon optimized nAChR hβ2 subunits but the current responses are higher from oocytes expressing the codon optimized hβ2 subunits

**Figure 3:**
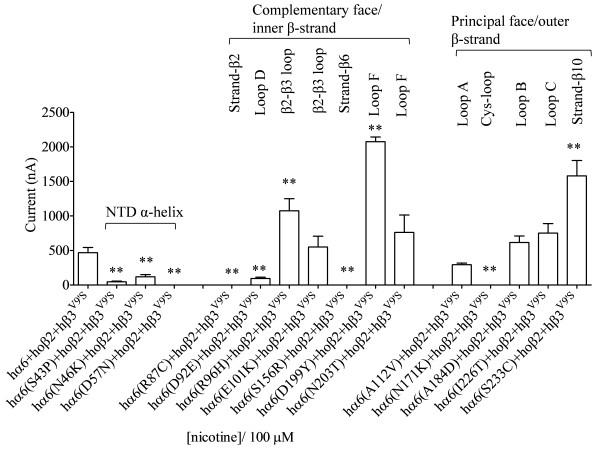
**Variations in nAChR hα6 subunit influence the current responses of human α6β2β3**^**V9’S**^**-nAChRs expressed in oocytes using codon optimized nAChR hβ2 subunits.** Mean (±SEM) peak inward current responses upon exposure to 100 μM nicotine (5 sec exposure; ordinate) are estimated from oocytes (n = 3-7) voltage clamped at −70 mV and heterologously expressing the indicated nAChR subunits. Current responses of hα6hβ2hβ3^V9’S^-nAChR are completely abolished (D57N or S156R), partially abolished (S43P, N46K, R87C, D92E or N171K), not changed (E101K, A112V, A184D, N203T or I226T) and increased (R96H, D199Y or S233C) as a result of the indicated variations in nAChR hα6 subunits. Oocytes coexpressing nAChR hα6(D199Y) subunits, codon optimized hβ2 subunits and hβ3^V9’S^ subunits yield largest current responses to nicotine. Comparisons of peak current responses between control (hα6hβ2hβ3^V9’S^-nAChR) and variant nAChR groups were analyzed using one-way ANOVA with Dunnett’s multiple comparisons test (*, p < 0.05; and **, p < 0.01).

**Figure 4:**
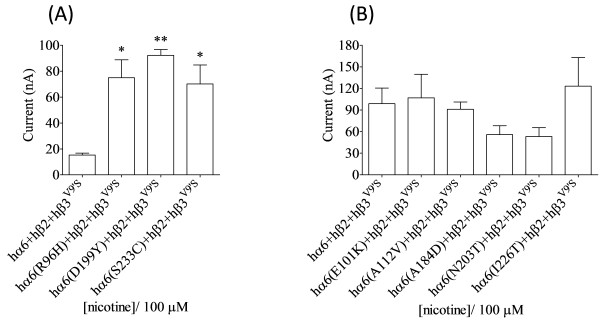
**Variations in nAChR hα6 subunit influence the current responses of human α6β2β3**^**V9’S**^**-nAChR expressed in oocytes using WT nAChR hβ2 subunits.** Attempts were made to verify the results obtained for human α6β2β3^V9’S^-nAChR using WT nAChR hβ2 subunits instead of codon optimized hβ2 subunits. Mean (±SEM) peak inward current responses upon exposure to 100 μM nicotine (5 sec exposure; ordinate) are estimated from oocytes (n = 3-7) voltage clamped at −70 mV and heterologously expressing the indicated nAChR subunits. **(A)** Oocytes coexpressing nAChR hα6^(R96H, D199Y or S233C)^, hβ2_WT_ and hβ3^V9’S^ subunits yield current responses to nicotine that are higher than those expressing nAChR hα6, hβ2_WT_ and hβ3^V9’S^ subunits. These results are in agreement with those obtained using codon optimized hβ2 subunits. **(B)** Initial recordings 3 days after cRNA injection indicated that oocytes expressing WT hα6 or variant hα6^(E101K, A112V, A184D, N203T or I226T)^ subunits along with WT hβ2 and hβ3^V9’S^ subunits yield ~10-30 nA of current in response to 100 μM nicotine [see the hα6hβ2hβ3^V9’S^-nAChR response in **(A)**]. However recordings done after 2 additional days of waiting indicated that nicotine elicited current responses from human α6^E101K^β2β3^V9’S^-, α6^A112V^β2β3^V9’S^-, α6^A184D^β2β3^V9’S^-, α6^N203T^β2β3^V9’S^- or α6^I226T^β2β3^V9’S^-nAChRs are equal (p > 0.05) to those obtained from human α6β2β3^V9’S^-nAChRs. These results are in agreement with those obtained using codon optimized hβ2 subunits. Comparisons between groups were analyzed using one-way ANOVA with Tukey’s post hoc comparison and only those differ from the control (hα6hβ2hβ3^V9’S^-nAChR) are shown with asterisks. *, p < 0.05; and **, p < 0.01.

Incorporation gain-of-function hβ3^V9’S^ subunits instead of WT hβ3 subunits lead to expression of functional hα6hβ2hβ3^V9’S^-nAChRs but their peak current responses are minimal [25]. In an earlier effort [26], this observation could not be achieved although the use of mouse (m) α6 subunit instead of hα6 subunit led to expression of functional mα6hβ2hβ3^V9’S^-nAChRs [26]. Nonetheless here we show that functional hα6hβ2hβ3^V9’S^-nAChRs could be expressed in oocytes by injecting higher amount of cRNAs (~23 ng) for each subunit. Furthermore the use of a codon optimized nAChR hβ2 subunit instead of a WT hβ2 subunit increases the current responses of human α6β2β3^V9’S^-nAChRs. Results (Figures 3 and 4) indicate that oocytes co-injected with hα6 subunit, codon optimized hβ2 subunit, and hβ3^V9’S^ subunit cRNAs elicit ~31-fold higher (15 ± 2 nA vs. 469 ± 75 nA; p < 0.05) current responses than those injected with hα6, hβ2 and hβ3^V9’S^ subunit cRNAs in response to activation by 100 μM nicotine. Therefore we decided to measure current responses from oocytes coexpressing variant-hα6 subunits, codon optimized hβ2 subunits and hβ3^V9’S^ subunits first and then verify these results using WT hβ2 subunits wherever it is feasible.

### Human α6β4- and α6β4β3-nAChRs are functional and current responses obtained from oocytes expressing α6β4β3-nAChRs are higher than those expressing hα6hβ4-nAChRs

**Figure 5:**
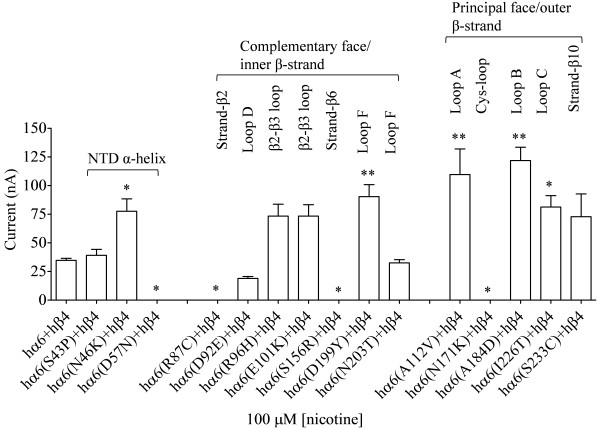
**Variations in nAChR hα6 subunit influence the current responses of hα6hβ4-nAChRs.** Mean (±SE) peak inward current responses upon exposure to 100 μM nicotine (5 sec exposure; ordinate) are estimated from oocytes (n = 6-9) voltage clamped at −70 mV and heterologously expressing the indicated nAChR subunits. Oocytes coexpressing hα6^A184D^ (variation in loop D) and hβ4 subunits yield largest current responses to 100 μM nicotine. Comparisons of peak current responses between control (hα6hβ4-nAChR) and variant groups were analyzed using one-way ANOVA with Dunnett’s multiple comparisons test (*, p < 0.05; and **, p < 0.01).

**Figure 6:**
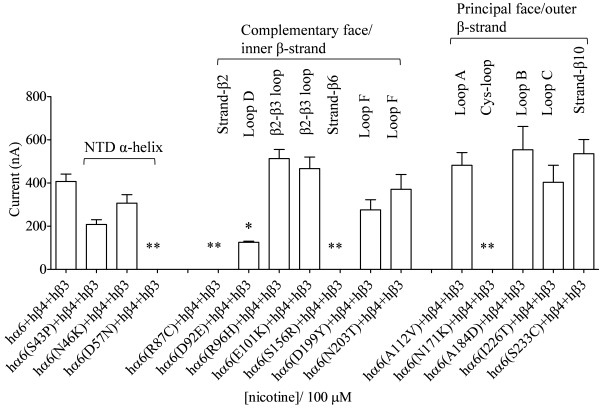
**Variations in nAChR hα6 subunit influence the current responses of hα6hβ4hβ3-nAChRs.** Mean (±SE) peak inward current responses upon exposure to 100 μM nicotine (5 sec exposure; ordinate) are estimated from oocytes (n = 6-11) voltage clamped at −70 mV and heterologously expressing the indicated nAChR subunits. Oocytes coexpressing hα6^(D57N, R87C, S156R or N171K)^ subunits plus hβ4 and hβ3 subunits do not yield current responses to nicotine though those coexpressing hα6, hβ4 and hβ3 subunit yield fairly robust current responses. hα6 subunit variation Asp92Glu (in loop D) partially abolishes the peak current responses of hα6hβ4hβ3-nAChRs. hα6 subunit variations Asn46Lys, Arg96His, Glu101Lys, Ala112Val, Ala184Asp, Asn203Thr, Ile226Thr or Ser233Cys do not affect nicotine elicited peak current responses of hα6hβ4hβ3-nAChRs. Comparisons between control (hα6hβ4hβ3) and variant groups were analyzed using one-way ANOVA with Dunnett’s multiple comparisons test (*, p < 0.05; and **, p < 0.01).

**Figure 7:**
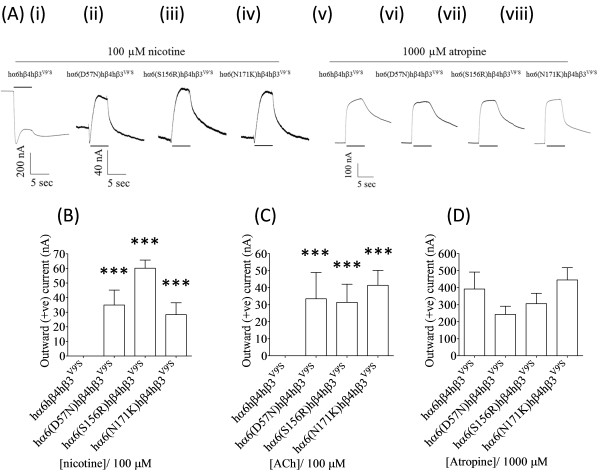
**Nicotinic agonists act as antagonists at hα6**^**(D57N, S156R or N171K)**^**hβ4hβ3**^**V9’S**^**-nAChRs. (A)** Representative traces are shown for inward or outward current responses from oocytes (voltage clamped at -70 mV) responding to the application of indicated concentrations of nicotine or atropine (shown with the duration of drug exposure as black bars above or below the traces) and expressing hα6hβ4hβ3^V9’S^- **[(A) (i) and (v)]**, hα6^D57N^hβ4hβ3^V9’S-^**[(A) (ii) and (vi)]**, hα6^S156R^hβ4hβ3^V9’S^**[(A) (iii) and (vii)]** or hα6^N171K^hβ4hβ3^V9’S^- **[(A) (iv) and (viii)]** nAChRs. Calibration bars are for 200 (i), 40 [(ii), (iii) and (iv)] or 100 [(v), (vi), (vii) and (viii)] nA currents (vertical) or 5 sec (horizontal). Results for these and other studies were used to estimate mean (±SE) peak outward current responses to 100 μM nicotine **(B)**, 100 μM ACh **(C)** or 1000 μM atropine **(D)** from oocytes (n=3-6) heterologously expressing the indicated nAChR subunits. hα6^(D57N,^^S156R^^or^^N171K)^hβ4hβ3^V9’S^-nAChRs elicit outward (positive) current in response to nicotine **(B)** or ACh **(C)** which is completely absent from hα6hβ4hβ3^V9’S^-nAChRs **[(B) and (C)]**. hα6^(D57N,^^S156R^^or^^N171K)^hβ4hβ3^V9’S^-nAChRs in rare occasion display minuscule inward currents in response to ACh or nicotine (data not shown). Comparisons between groups were analyzed using one-way ANOVA with Tukey’s post hoc comparison and only those differ from the control (hα6hβ4hβ3^V9’S^-nAChR) are marked with asterisks. ***; p < 0.001.

While hα6hβ4- and hα6hβ4hβ3- nAChRs are difficult to express [25,26], functional hybrid nAChR consisting of mα6, hβ4 and hβ3 subunits (i.e., mα6hβ4hβ3-nAChR) [26] and others such as human α2β4-, α3β2-, α3β4-, α4β2- or α4β4-nAChRs could be easily expressed in oocytes using a relatively lower amount (~1-4 ng) of cRNA for each subunit [36]. In this study we used relatively larger amount of cRNA (~23 ng) for each subunit for functional expression of hα6hβ4- or hα6hβ4hβ3-nAChRs. This approach has been shown to result in functional expression of hα6hβ4*-nAChRs [29]. Like reported previously [29], here we observed hα6hβ4- and hα6hβ4hβ3-nAChRs are functional and the peak current responses of hα6hβ4hβ3-nAChRs exceed those of hα6hβ4-nAChRs (35 ± 2 nA vs. 407 ± 34 nA in response to 100 μM nicotine, ~12 -fold increase, p < 0.05; and 25 ± 3 nA vs. 502 ± 60 nA in response to 1000 μM ACh, ~20-fold increase, p < 0.05), demonstrating a potentiation effect of hβ3 subunits on the peak current responses of hα6hβ4-nAChRs (Figures 5–6 and 7; Table 3 and Additional file 1: Figure S1 and Table S1). The EC_50_ values for nicotine and ACh acting at hα6hβ4hβ3-nAChR is determined to be 12 μM and 40 μM respectively (Tables 3 and Additional file 1: S1). Lack of current responses at lower concentrations of nicotine or ACh precluded our ability to construct CR curves for hα6hβ4-nAChRs.

**Table 3:** Parameters for ACh action at WT or variant hα6hβ4*- nAChRs

	**Potency**			**Peak response**		
**nAChR subunit combinations**	**n**	**EC**_**50 **_**(μM) (95% CI)**	**n**_**H **_**± SE n**_**H**_	**n**	**Mean I**_**max **_**± SE (nA)**	**I**_**max **_**conc. (μM)**
hα6 + hβ4		18^1^	-	5	30 ± 3	1000
hα6(R96H) + hβ4	3	-	-	3	31 ± 4	1000
hα6(A184D) + hβ4	3	11 (9.3-13)	1.3 ± 0.16	5	86 ± 5 ⬆	1000
hα6(D199Y) + hβ4	3	16 (14–19)	1.1 ± 0.04	5	73 ± 12⬆	1000
hα6(S233C) + hβ4	3	14 (11–17)	0.95 ± 0.08	4	90 ± 23	1000
hα6 + hβ4 + hβ3	4	40 (29–56)	1.1 ± 0.1	4	502 ± 60▲	1000
hα6(R96H) + hβ4 + hβ3	3	21 (17–26) ⬇	0.96 ± 0.07	6	905 ± 146▲	1000
hα6(A184D) + hβ4 + hβ3	3	17 (14–22) ⬇ ▲	0.97 ± 0.04	7	788 ± 202▲	1000
hα6(D199Y) + hβ4 + hβ3	3	24 (20–28) ⬇ ▲	1.1 ± 0.07	7	494 ± 51▲	1000
hα6(S233C) + hβ4 + hβ3	3	16 (12–21) ⬇	0.98 ± 0.1	3	771 ± 150▲	1000

^1^From Kuryatov *et al*. (2000).

**Figure 8:**
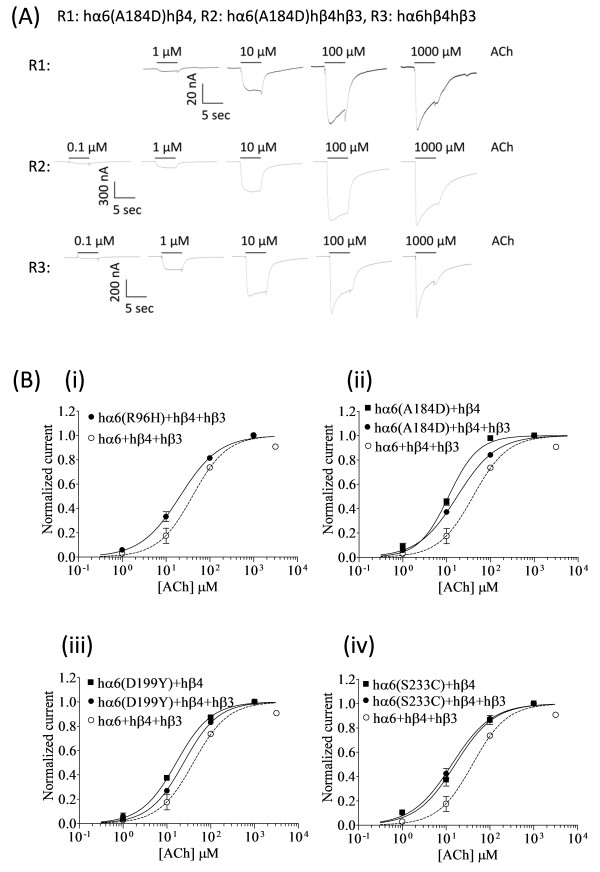
Variations in nAChR hα6 subunit influence the ACh sensitivity of hα6hβ4*-nAChRs. (A) Representative traces are shown for current responses from oocytes (voltage clamped at −70 mV) responding to the application of indicated concentrations of ACh (shown with the duration of drug exposure as black bars above the traces) and expressing indicated nAChR (i.e., R1: hα6^A184D^hβ4-nAChR, R2: hα6^A184D^hβ4hβ3-nAChR, R3: hα6hβ4hβ3-nAChR]. **(B)** Results averaged across experiments were used to produce concentration-response (CR) curves (ordinate-mean normalized current ± SEM; abscissa - ligand concentration in log μM) for inward current responses to ACh as indicated for the nAChR expressed in oocytes and voltage clamped at −70 mV. Current amplitudes are represented as a fraction of the peak inward current amplitude in response to the most efficacious concentration of ACh. Leftward shifts in ACh CR curves for hα6^R96H^hβ4hβ3-(●) **[(B) (i)]**, hα6^A184D^hβ4hβ3-(●) **[(B) (ii)]**, hα6^D199Y^hβ4hβ3-(●) **[(B) (iii)]**, or hα6^S233C^hβ4hβ3-(●) **[(B) (iv)]** nAChR are evident relative to that of hα6hβ4hβ3-nAChR (○). Furthermore ACh curves for hα6^A184D^hβ4-(■) **[(B) (ii)]**, hα6^D199Y^hβ4-(■) **[(B) (iii)]**, or hα6^S233C^hβ4-(■) **[(B) (iv)]** nAChR are shifted leftward relative to those nAChR containing the same subunits but in the additional presence of hβ3 subunits. See Table 3 for parameters of ACh action.

Potencies [micromolar EC_50_ values with 95% confidence intervals (CI)], Hill coefficients (n_H_ ± SE), mean ± SE peak response (I_max_ in nanoamps) and concentrations (μM) where maximal peak current amplitudes (I_max_) achieved are provided for ACh acting at nAChR composed of the indicated subunits and from the indicated number of independent experiments (n) based on studies as shown in Figure 8. ⬆ or ⬇ indicate a significant (p < 0.05) increase or decrease in indicated parameter at the indicated nAChR subtype relative to nAChR containing the same subunits but in the presence of the WT hα6 subunit (i.e., hα6hβ4- vs. variant-hα6hβ4- nAChR; hα6hβ4hβ3- vs. variant-hα6hβ4hβ3- nAChR). ▲ or ▼ indicate a significant (p < 0.05) increase or decrease, respectively, in indicated parameter at the indicated nAChR subtype relative to nAChR containing the same subunits but in the absence WT β3 subunits (i.e., hα6hβ4- vs. hα6hβ4hβ3- nAChR; variant-hα6hβ4- vs. variant-hα6hβ4hβ3- nAChR). ‘-‘ indicates that inconsistent functional responses in two electrode voltage clamp studies precluded determination of the parameter of interest.

### Variations in nAChR hα6 subunit influence the current responses of human α6β2β3^V9’S^-nAChRs but they do not alter the null responses observed from oocytes coexpressing nAChR hα6 and hβ2 (WT or codon optimized) subunits in the presence or absence of hβ3 subunits

We sought to determine whether coexpression of the variant nAChR hα6 subunits with WT or codon optimized hβ2 subunits in the presence or absence of hβ3 subunits would result in expression of functional nAChR in oocytes. We did not observe detectable current responses from these oocytes except that coexpression of codon optimized hβ2 and hβ3 subunits with either hα6^D199Y^ or hα6^S233C^ subunits appeared to result in functional nAChRs but their peak current responses to 100 μM nicotine (in the range of 10 to 20 nA) could not be reliably and consistently measured (data not shown). However, oocytes coexpressing nAChR hα6, hα6^S43P^, hα6^N46K^, hα6^D92E^, hα6^R96H^, hα6^E101K^, hα6^A112V^, hα6^A184D^, hα6^D199Y^, hα6^N203T^, hα6^I226T^ or hα6^S233C^ subunits with codon optimized hβ2 subunits and gain-of-function hβ3^V9’S^ subunits responded to the application of 100 μM nicotine with peak currents (I_max_, mean ± SE) of 469 ± 75 nA, 47 ± 9 nA, 118 ± 33 nA, 66 ± 20 nA, 1075 ± 175, 551 ± 156 nA, 293 ± 24 nA, 616 ± 95 nA, 2076 ± 68 nA, 762 ± 253 nA, 753 ± 137 nA or 1582 ± 221 nA, respectively (Figure 3). Most of the oocytes expressing nAChR hα6^D57N^, hα6^R87C^, hα6^S156R^ or hα6^N171K^ subunits along with codon optimized hβ2 subunits and hβ3^V9’S^ subunits yield null responses to nicotine although on occasion, upon repeated attempts, some of these oocytes yield 10–30 nA of current in responses to 100 μM nicotine. Therefore variations Asp57Asn, Arg87Cys, Ser156Arg or Asn171Lys abolish; variations Ser43Pro or Asn46Lys reduce (75-93%; p < 0.05); variations Asp92Glu, Glu101Lys, Ala112Val, Ala184Asp, Asn203Thr or Ile226Thr do not affect (p > 0.05); and variations Arg96His, Asp199Tyr or Ser233Cys increase (p < 0.05; ~2-5 fold) the peak current responses of hα6hβ2hβ3^V9’S^-nAChRs.

We attempted to reproduce these results using WT hβ2 subunits. Oocytes coexpressing nAChR hα6^D57N^, hα6^R87C^, hα6^S156R^, hα6^N171K^, hα6^S43P^, hα6^N46K^ or hα6^D92E^ subunits along with WT hβ2 subunits and hβ3^V9’S^ subunits did not yield current responses to nicotine. These results conversely indicate that a null response observed for hα6^(S43P, N46K^^or^^D92E)^hβ2hβ3^V9’S^-nAChRs using WT hβ2 subunits is not truly null as some degree of function is detected using the codon optimized hβ2 subunits. Hence a null function detected using WT hβ2 subunits in fact is a level of current response that is below our limit of detection.

This prompted us to evaluate current responses from oocytes coexpressing nAChR hα6^R96H^, hα6^D199Y^ or hα6^S223C^ subunits in conjunction with WT hβ2 and hβ3^V9’S^ subunits as these three variants expressed in oocytes in the presence of codon optimized hβ2 and hβ3^V9’S^ subunits elicit largest responses to nicotine (Figure 3). Surprisingly, there was emergence of (hα6^R96H, D199Y or S223C^)hβ2hβ3^V9’S^-nAChRs and their peak current responses to 100 μM nicotine were higher (p < 0.05; ~2-5 fold) than those of hα6hβ2hβ3^V9’S^-nAChRs [Figure 4A].

The (hα6, hα6^E101K^, hα6^A112V^, hα6^A184D^, hα6^N203T^ or hα6^I226T^)hβ2hβ3^V9’S^-nAChRs expressed in oocytes 3 days after injection yielded ~15-30 nA (data not shown) current in responses to 100 μM nicotine. In order to confirm that these current responses are real and due to the expression of functional nAChRs, recordings were done after waiting for additional 2 days. This time peak current responses could be reliably measured [Figure 4B]. Mean (±SEM) level of current responses for these nAChR ranged from 53 ± 12 nA (for hα6^N203T^hβ2hβ3^V9’S^-nAChR) to 123 ± 40 nA [for hα6^I226T^β2β3^V9’S^-nAChR; Figure 4B]. However, they were similar (p > 0.05) to those of the control. Hence coexpression with WT hβ2 subunits instead of codon optimized hβ2 subunits did not alter the outcome. Note that the current responses of hα6hβ2hβ3^V9’S^-nAChRs to nicotine were assayed 5 days after injection (but not 3 days after injection as shown for Figure 4A]. Hence there is disparity in peak current responses of hα6hβ2hβ3^V9’S^-nAChR in the two panels of Figure 4.

### Variations in nAChR hα6 subunit influence the current responses of hα6hβ4-nAChRs

Oocytes coexpressing nAChR hα6, hα6^S43P^, hα6^N46K^, hα6^D92E^, hα6^R96H^, hα6^E101K^, hα6^A112V^, hα6^A184D^, hα6^D199Y^, hα6^N203T^, hα6^I226T^ or hα6^S233C^ subunits with hβ4 subunits responded to the application of 100 μM nicotine with peak currents (I_max_, mean ± SE) of 35 ± 2 nA, 39 ± 5 nA, 78 ± 11 nA, 19 ± 2 nA, 73 ± 10 nA, 73 ± 10 nA, 110 ± 22 nA, 122 ± 12 nA, 90 ± 11 nA, 33 ± 3 nA, 81 ± 10 nA or 73 ± 20 nA, respectively (Figure 5). Predominantly null responses were observed from oocytes coexpressing nAChR hα6^D57N^, hα6^R87C^, hα6^S156R^ or hα6^N171K^ subunits and hβ4 subunits. However, occasionally some of these oocytes seemed to yield 10–20 nA of current in responses to 100 μM nicotine. Hence current responses of hα6hβ4-nAChRs were increased (p < 0.05; ~2-3-fold) as a result of Asn46Lys, Ala112Val, Ala184Asp, Asp199Tyr or Ile226Thr variations and compromised (p < 0.05; ~100%) due to Asp57Asn, Arg87Cys, Ser156Arg or Asn171Lys variations in hα6 subunit. Also current responses to 1000 μM ACh were increased (p < 0.05) from oocytes coexpressing nAChR hα6^A184D^ or hα6^D199Y^ subunits and hβ4 subunits relative to those coexpressing hα6 and hβ4 subunits (Table 3).

### Variations in nAChR hα6 subunit influence the current responses of human α6β4β3-nAChRs

Mean (±SE) peak current responses obtained from oocytes expressing nAChR hβ3 and hβ4 subunits in the presence of nAChR hα6, hα6^S43P^, hα6^N46K^, hα6^D92E^, hα6^R96H^, hα6^E101K^, hα6^A112V^, hα6^A184D^, hα6^D199Y^, hα6^N203T^, hα6^I226T^ or hα6^S233C^ subunits were 407 ± 34 nA, 208 ± 22 nA, 307 ± 39 nA, 126 ± 5 nA, 513 ± 42 nA, 467 ± 54 nA, 482 ± 59 nA, 554 ± 108 nA, 276 ± 47 nA, 371 ± 68 nA, 403 ± 79 nA or 536 ± 66 nA, respectively (Figure 6). Oocytes expressing hα6^D57N^, hα6^R87C^, hα6^S156R^ or hα6^N171K^ subunits in the presence of nAChR hβ4 and hβ3 subunits did not elicit current responses to nicotine (data not shown). Hence additional incorporation of hβ3 subunits into (hα6, hα6^S43P^, hα6^N46K^, hα6^D92E^, hα6^R96H^, hα6^E101K^, hα6^A112V^, hα6^A184D^, hα6^D199Y^, hα6^N203T^, hα6^I226T^ or hα6^S233C^)hβ4-nAChR complexes, but not into putative (hα6^D57N^, hα6^R87C^, hα6^S156R^ or hα6^N171K^)hβ4-nAChR complexes, resulted in potentiated (3–12 fold; p < 0.05) current responses from oocytes (see Figures 5 and 6). These former results are consistent with the observation that incorporation of hβ3 subunits potentiates the peak current responses of hα6hβ4-nAChRs [29]. Therefore variations Asp57Asn, Arg87Cys, Ser156Arg or Asn171Lys abolish (p < 0.01); variation Asp92Glu reduces (p < 0.05); and variations Asn46Lys, Arg96His, Glu101Lys, Ala112Val, Ala184Asp, Asp199Tyr, Asn203Thr, Ile226Thr or Ser233Cys do not affect (p > 0.05) the peak current responses of hα6hβ4hβ3-nAChRs. Also variations Arg96His, Asp199Tyr, Ala184Asp or Ser233Cys in hα6 subunit do not affect (p > 0.05) the ACh (1000 μM) induced current responses of hα6hβ4hβ3-nAChRs (Table 3).

### Further studies on Asp57Asn, Ser156Arg and Asn171Lys variations reveal that nicotine, ACh or atropine acts as antagonists at hα6^D57N^hβ4hβ3^V9’S^, hα6^S156R^hβ4hβ3^V9’S^ and hα6^N171K^hβ4β3^V9’S^-nAChRs

In order to further understand the bases for the null effects of Asp57Asn, Arg87Cys, Ser156Arg and Asn171Lys variations in nAChR hα6 subunit, we coexpressed nAChR hα6, hα6^D57N^, hα6^R87C^, hα6^S156R^ or hα6^N171K^ subunits with hβ4 and gain-of-function hβ3^V9’S^ subunits and current responses from these oocytes to nicotine (100 μM), ACh (100 μM) or atropine (1000 μM) were measured. As expected hα6hβ4hβ3^V9’S^-nAChRs yielded robust inward current in response to activation by nicotine or ACh and outward reversible currents in response to blockade of spontaneously opening channels by atropine (Figure 8) [26-28,36]. However, hα6^D57N^hβ4hβ3^V9’S^, hα6S^156R^hβ4hβ3^V9’S^ or hα6^N171K^hβ4β3^V9’S^-nAChRs expressed in oocytes produced outward reversible currents in response to ACh, nicotine or atropine (Figure 8). Such outward reversible currents were not observed from oocytes coexpressing hα6, hα6^D57N^, hα6^S156R^ or hα6^N171K^ subunits with hβ2 and hβ3^V9’S^ subunits (data not shown). These results indicate that hβ3^V9’S^ (a surrogate for hβ3) subunits in fact integrates into hα6^D57N^hβ4, hα6^S156R^hβ4 or hα6^N171K^hβ4-nAChR complexes. Apparently these cell surface expressed and spontaneously opening nAChRs are not responsive to agonist action of nicotine or ACh but it seems ACh, nicotine and also atropine are acting as antagonists at these nAChRs. Current responses to ACh, nicotine or atropine were not obtained from oocytes coexpressing nAChR hβ4 or hβ2 subunits and hα6^R87C^ and hβ3^V9’S^ subunits (Figure 2).

### Variations in nAChR hα6 subunits that affect the agonist (nicotine or ACh) sensitivity of hα6hβ4*-nAChRs

Concentration-response (CR) curves for WT or variant hα6hβ4*-nAChR were produced, wherever feasible, in a manner to glean maximum comparative information about them. As we could not produce CR curves for hα6hβ4-nAChRs, for comparative analysis, we have adopted the EC_50_ values for nicotine (7.1 μM) and ACh (18 μM) acting at human α6β4-nAChRs reported by Kuryatov *et al*. (2000). EC_50_ values generated for other variant hα6hβ4*-nAChRs are provided in Tables 3 and Additional file 1: Table S1. Results indicated that nicotine EC_50_ value at hα6^D199Y^hβ4-nAChRs and ACh EC_50_ values at hα6^(A184D^^or^^D199Y)^hβ4-nAChRs are lower (~1.5-2.5 fold; p < 0.05) than those of corresponding nAChRs additionally incorporating hβ3 subunits. Furthermore nicotine EC_50_ values at hα6^(A184D or S233C)^hβ4hβ3-nAChRs and ACh EC_50_ values at hα6^(R96H, A184D, D199Y or S233C)^hβ4hβ3-nAChRs are also lower (~1.7-2.5 fold; p < 0.05) than those of hα6hβ4hβ3-nAChRs. Hence nicotine or ACh sensitivity of hα6hβ4- and/or hα6hβ4hβ3- nAChRs are marginally or significantly increased as a result of Arg96His, Ala184Asp, Asp199Tyr or Ser233Cys variations in hα6 subunit (Figure 8 and Additional file 1: Figure S1, Tables 3 and Additional file 1: Table S1). These results, in general, indicate that incorporation of nAChR hβ3 subunit into WT or variant hα6hβ4-nAChR complexes result in marginally lower EC_50_ values for nicotine or ACh.

## Discussion

In discussion of our results we presume that empirical changes in peak current levels and/or sensitivity of nAChRs are indicative of successful incorporation of WT or mutant β3 subunits into functional WT or variant hα6*-nAChRs. However, in the absence of data for the effect of the variations in nAChR hα6 subunit on subunit biogenesis and trafficking; receptor assembly and level of cell surface expression; ligand binding and channel open probability; etc. we have relied on cell surface functional receptors to draw inferences, conclusions and/or propose additional hypotheses about functional expression of WT or variant hα6*-nAChRs.

The inability to express functional hα6hβ2*-nAChRs without use of gain-of-function hβ3 subunits (i.e., hβ3^V9’S^) confounds our ability to make inferences about assembly of the WT subunits but it gives credence to the idea that nAChR hβ3 subunits exert dominant negative effect on the function of α6β2*-nAChRs [25]. Alternatively, there possibly exists an undetectable level of basal function for hα6hβ2hβ3-nAChRs that gets amplified upon substitution of hβ3^V9’S^ subunits for hβ3 subunits. The lack of function for heterologously expressed hα6hβ2- and hα6hβ2hβ3-nAChRs additionally could be indicative of lack of presence of other nAChR subunits (e.g., α3 or α4), chaperones or cellular components that typically would facilitate assembly and functional expression of α6β2*-nAChRs in neurons or other cells [26,27,29]. Nonetheless in this study we have successfully used hα6hβ2hβ3^V9’S^-nAChRs as a model to evaluate the effect of variations in hα6 subunit on function of hα6hβ2*-nAChRs. Our results are greatly enhanced by the use of a codon optimized nAChR hβ2 subunit and advantages of use of such codon-optimized nAChR subunits were demonstrated previously [27,28,37,38].

The current results and experimental approach indicate that hβ3 subunits do not exert a dominant negative effect on the function of hα6hβ4*-nAChRs [25-28]. Rather they promote its functional expression as is shown here and elsewhere [29]. It appears that hβ3 subunits need a consistent basal level of expression of nAChR hα6 and hβ4 subunits, manifested as consistent functional expression of hα6hβ4-nAChRs, for their integration and promotion of functional expression of the resultant nAChRs. hα6hβ4-nAChRs expressed using 1 ng or similar amount of cRNAs for each subunit, typical for functional expression of many other nAChR subtypes [36], are barely detected functionally on cell surface. Further microinjection of hβ3 subunits in similar amounts or in 20-fold excess of other α and β subunits [25,26] seems not to affect the outcome but upon substitution of hβ3^V9’S^ subunits for hβ3 subunits there is emergence of highly functional hα6hβ4hβ3^V9’S^-nAChRs. Remember large excess of hβ3 subunits might be promoting non-functional, dead-end intermediates [39] exacerbating the already poor expression of nAChR hα6 and hβ4 subunits or poor functional expression of hα6hβ4-nAChRs leading to the notion that hβ3 subunits exert a dominant negative effect on the function of hα6hβ4*-nAChRs. The increased functionality of hα6hβ4hβ3-nAChRs relative to hα6hβ4-nAChRs, rather than a null or decreased functionality [25], is analogous to studies using chimeric (α6/α3) subunits (containing the N-terminal domain of the nAChR α6 subunit substituting for that of the otherwise α3 subunit) instead of WT α6 subunits that shows a potentiating effects of WT β3 subunit on function of (α6/α3)(β2 or β4)*-nAChRs [29].

Consistent with the notion that oocytes expressing hα6hβ4hβ3-nAChRs yield higher I_max_ than those expressing hα6hβ4-nAChRs, hα6^(S43P, N46K, D92, R96H, E101K, A112V, A184D, D199Y, N203T, I226T or S233C)^hβ4hβ3- nAChRs expressed in oocytes yield higher peak current responses than those of their binary nAChR counterparts lacking the hβ3 subunits. However, there is not a potentiation effect on current responses upon incorporation of hβ3 subunits into presumed hα6^(D57N, R87C, S156R or N171K)^hβ4- nAChR complexes. The lack of current responses from oocytes expressing hα6^(D57N, R87C, S156R or N171K)^hβ4hβ3-nAChRs, wherein hα6hβ4- and hα6hβ4hβ3-nAChRs are functional, is probably not indicative of exertion of dominant negative effect by nAChR hβ3 subunits. It appears that these variations in nAChR α6 subunit destroy the ability of participating subunits to assemble into a typical functional nAChR. This is because hα6^(D57N, S156R or N171K)^hβ4hβ3^V9’S^-nAChRs display outward (positive) reversible current in responses to nicotine or ACh defying the expectation that they would display inward current responses like those typically seen with hα6hβ4hβ3^V9’S^-nAChRs [26-28]. Also hα6^(D57N, S156R or N171K)^hβ4hβ3^V9’S^-nAChRs elicit outward (positive) reversible current to atropine, a characteristic that is also shared by spontaneously opening hα6hβ4hβ3^V9’S^- or other β4β3*-nAChRs [26-28,36] indicating that both nicotine and ACh are acting as antagonists than as agonists at these nAChRs. The antagonism of ACh or nicotine (and that of atropine) appears to be due to the blockade of spontaneously opening hα6^(D57N, S156R, or N171K)^hβ4hβ3^V9’S^-nAChRs.

Lack of consistent and reproducible current responses from oocytes coexpressing hα6^(D57N, R87C, S156R or N171K)^ subunits with hβ2 and hβ3^V9’S^ subunits; or with hβ4 subunits probably indicate that these variations, respectively, presumably in the N-terminal α-helix, strand β2, strand β6 and cysteine-loop disrupt assembly of the subunits into a typical functional pentamer in a manner similar to those encountered with hα6^(D57N, R87C, S156R or N171K)^hβ4hβ3- nAChRs. The Asn^171^ residue in hα6 subunit, mutated to Lys (K), is a potential target for N-glycosylation. Potential de-glycosylation of the nAChR hα6 subunit as a result of the Asn171Lys variation could affect stability and trafficking of hα6(N171K)hβ2hβ3^V9’S^-, hα6(N171K)hβ4-, hα6(N171K)hβ4hβ3- or hα6(N171K)hβ4hβ3^V9’S^-nAChRs to the cell surface [40,41]. The outward current responses of hα6^N171K^hβ4hβ3^V9’S^-nAChR to ACh or nicotine, in contrast to an expected inward current, probably corroborate this point. Also the Asn171Lys variation in hα6 subunit could disrupt the essential role of the cys-loop in coupling agonist (e.g., nicotine) binding to channel gating (opening) in hα6hβ2*-, hα6hβ4- or hα6hβ4hβ3- nAChRs. Note that Asp^57^, Arg^87^, Ser^156^ and Asn^171^ residues of hα6 subunit are strongly conserved in nAChR subunits from various organisms indicating that a variation in these residues could not be tolerated without negative consequences on the structure-function relationship of hα6*-nAChRs. Additionally it appears elimination of a positively charged residue at AA position 87 (Arg87Cys) or introduction of a positively charged residue at position 171 (Asn171Lys) is having negative functional consequences.

For oocytes expressing hα6^(S43P, N46K or D92E)^hβ2hβ3^V9’S^-, hα6^D92E^hβ4- or hα6^D92E^hβ4hβ3-nAChRs to display compromised or reduced current responses could be due to decreased cell surface functional expression of these receptors. Additionally it could be due to change in inherent structure of the receptor molecules because of the indicated AA variations. It appears that the Asp^92^ residue, strongly conserved across various nAChR subunits located in loop D (β2-β3 loop) in the complementary face of the hα6 subunit, upon mutation to a glutamate (that results in an increase in AA side chain length) is having a negative effect on the subunit assembly of α6β2β3^V9’S^-, α6β4-, or α6β4β3- nAChRs possibly by affecting the subunit interaction at the positive (+) face of the β2 or β4 subunit and negative (−) face of the α6 subunit [i.e., β2 or β4 (+):(−)α6]; and/or at the positive (+) face of the β3 or β3^V9’S^ subunit and the negative (−) face of the α6 subunit [i.e., β3 or β3^V9’S^(+):(−)α6] [Figure 2(C), (D) and (E)]. The reduction in current responses of hα6hβ2hβ3^V9’S^-nAChRs but not those of hα6hβ4- or hα6hβ4hβ3-nAChRs as a result of Ser43Pro variation in hα6 subunit could be due to a subtype specific effect of the variation. Similarly a reduction in current responses of hα6hβ2hβ3^V9’S^-nAChRs; no change in current responses of hα6hβ4hβ3-nAChRs; and an increase in current responses of hα6hβ4-nAChRs as a result of Asn46Lys variation in hα6 subunit could be a subtype specific effect of the variation. This in turn could be attributed to the introduction of a positively charged residue (i.e., Lys) at AA position 46 in hα6 subunit.

Variations in nAChR hα6 subunit that occur at residue 101 (Glu101Lys: β2-β3 loop/loop D), 112 (Ala112Val: loop A), 184 (Ala184Asp: loop B), 203 (Asn203Thr: loop F) or 226 (Ile226Thr: loop C) do not affect the peak current responses of hα6hβ2hβ3^V9’S^- or hα6hβ4hβ3-nAChRs, an indication that natural AA substitutions at these positions do not grossly affect the assembly, cell surface expression and/or structure-function relationship of these nAChRs. However, variations in loops A (Ala112Val), B (Ala184Asp) or C (Ile226Thr) in hα6 subunit substantially increases current responses from minimally functional hα6hβ4-nAChRs signifying the emerging notion that N-terminal loop residues are important in the assembly and functional expression of hα6*-nAChRs [26-28]. Also it is of significance that some of these variations in various loop residues are innocuous or beneficial for the functional expression of hα6hβ2hβ3^V9’S^-, hα6hβ4- or hα6hβ4hβ3-nAChRs an indication that these substitutions are tolerated in regions that are crucial in ligand binding and/or subunit assembly. Coincidentally hα6 subunits have non-conserved AAs at position 101 (Glu), 112 (Ala), 184 (Ala) or 226 (Ile); and a weakly conserved AA at position 203 (Asn) in an alignment analyses of human nAChR α subunits (see Figure 1). A weak or nonconserved AA residue probably indicates, but not necessarily always, a tolerance for these substitutions. However, such a broader interpretation becomes difficult to generalize as hα6 subunit seems to have strongly conserved AAs at positions 101 (Glu), 112 (Ala), 184 (Ala) and 226 (Ile) and a weakly conserved AA at position 203 (Asn) in an alignment analysis of α6 subunits from a limited number of other species.

Variations that occur in the nAChR hα6 subunit at AA residues 96 (Arg96His: β2-β3 loop/loop D), 199 (Asp199Tyr: loop F) or 233 (Ser233Cys: strand β10) increases the peak current responses from oocytes expressing hα6hβ2hβ3^V9’S^-nAChRs. However, these variations except Asp199Tyr do not have any effect on the peak current responses of hα6hβ4- or hα6hβ4hβ3-nAChRs expressed in oocytes again demonstrating a subtype specific effect of these variations in β2-β3 loop/loop D, loop F or β10-strand of hα6 subunit. It appears that the Arg^96^ or Asp^199^ residue located in the complementary face of the hα6 subunit is exerting a positive effect on the subunit assembly of α6β2β3^V9’S^- and α6β4-nAChRs possibly by affecting the subunit interaction at the positive (+) face of the β2 or β4 subunit and negative (−) face of the α6 subunit [i.e., β2 or β4 (+):(−)α6] ; and/or at the positive (+) face of the β3 or β3^V9’S^ subunit and the negative (−) face of the α6 subunit [i.e., β3 or β3^V9’S^(+):(−)α6] [Figure 2(C), (D) and (E)]. These results are similar to previously described reports that AA residue (e.g., Asn143: loop E) located in the negative face of the hα6 subunit influence the functional expression of hα6hβ2hβ3^V9’S^-nAChRs [26].

In the absence of data for WT hα6hβ4-nAChRs comparisons of EC_50_ values among WT and variant hα6hβ4-nAChRs remained incomplete. However, it is reported [29] that ACh (18 ± 5 μM) or nicotine (7.1 ± 2.6 μM) EC_50_ values at hα6hβ4-nAChRs are marginally lower than those of respective hα6hβ4hβ3-nAChRs (ACh EC_50_: 33 ± 8 μM and nicotine EC_50_:10 ± 3 μM). Our results indicate that this directionality in change in potency is preserved for hα6^(A184D, D199Y or S233C)^hβ4- nAChRs indicating that EC_50_ values at hα6^(A184D, D199Y or S233C)^hβ4-nAChRs is lower than those containing the same subunits but also additionally containing nAChR hβ3 subunits. But note that the increase in agonist sensitivity of nAChRs as a result of the variations in loop B (Ala184Asp), loop F (Asp199Tyr) and β strand 10 (Ser233Cys) (which connect to the trans-membrane domain I) in hα6 subunit are nominal implying that there could be subtle changes in receptor structures.

In the final analyses it is incumbent upon us to know the significance, if any, of these variations in hα6 subunit in the etiology of nicotine dependence and/or other hα6*-nAChR involved diseases. We would not know them until currently available tools, statistical or biotechnological, becomes mainstream. Specifically analyses of rare variations (i.e., Asp57Asn, Arg87Cys, Ser156Arg or Asn171Lys) in nAChR hα6 subunit that compromise the function of hα6*-nAChRs would be of prime interest in epidemiological or *in vivo* studies. Nonetheless individuals displaying altered hα6*-nAChR pharmacology as a result of rare variation in nAChR hα6 subunit are expected to exhibit differential responses to smoked nicotine.

## Conclusions

Our results presented here are in general agreement with the accumulated evidences that changes/mutations in loop residues and other structural residues could affect cell surface expression, assembly, structure and/or function of various nAChRs. Specifically N-terminal α-helix (Asp^57^); complementary face/inner β-fold (Arg^87^ or Asp^92^) and principal face/outer β-fold (Ser^156^ or Asn^171^) residues in hα6 subunit are crucial for functional expression of hα6hβ2*-, hα6hβ4- and hα6hβ4hβ3-nAChRs and natural variations in them (i.e., Asp57Asn, Arg87Cys, Asp92Glu, Ser156Arg or Asn171Lys) compromises their function. Additionally Ser^43^ or Asn^46^ (N-terminal α-helix) residues in hα6 subunit are important for functional expression of hα6hβ2*-nAChRs and natural variations in them (i.e., Ser43Pro or Asn46Lys) compromise its function. However, natural variations indicate Arg^96^ (β2-β3 loop/loop D), Asp^199^ (loop F) or Ser^233^ (β10-strand) residues in hα6 subunit could be taken advantage for promoting functional expression of hα6hβ2*-nAChRs. Similarly residues in N-terminal α-helix (Asn^46^), loop A (Ala^112^), loop B (Ala^184^), loop F (Asp^199^) or loop C (Ile^226^) could be substituted with their respective natural variations (i.e., Asn46Lys, Glu101Lys, Ala112Val, Ala184Asp, Asp199Tyr or Ile226Thr) for increased functional expression of poorly functional/expressed hα6hβ4-nAChRs. Thus, by studying natural variations in nAChR hα6 subunit, we have mapped AA residues in nAChR hα6 subunit important for cell surface functional expression of various subtypes of hα6*-nAChRs. These novel sites in nAChR hα6 subunit could be of promising use for creation of functional cell lines that could be helpful for drug screening; and development of new drug candidates selective for hα6*-nAChRs. This is of increasing importance given the potentially important roles for α6*-nAChRs in movement and movement disorders, mood disorders, and drug dependence [12,19,42-44].

## Methods

### Bioinformatics analyses

Human nAChR α (α1- α7, α9, α10) subunits or nAChR α6 subunits from various organisms were aligned using ClustalW and then edited for the purposes of presentation (Figure 1). A homology model of the human nAChR α6 subunit modeled on the 3-D coordinates of the muscle nAChR α subunit of *Torpedo marmorata* (PDB: 2BG9.A) [2] was retrieved using SWISS-MODEL [45] protein modeling server; and subsequently was rendered using UCSF Chimera (http://www.cgl.ucsf.edu/chimera/), a program for interactive visualization and analysis of molecular structures (Figure 2).

### Chemicals

All chemicals used in electrophysiology were obtained from Sigma Chemical Co. (St. Louis, MO, USA) except that L-nicotine was obtained from Arcos Organics (New Jersey, USA). Fresh agonist (acetylcholine or nicotine) and antagonist (atropine) stock solutions were made daily or diluted from frozen stock in Ringer's solution (OR2) which consisted of (in mM) 92.5 NaCl, 2.5 KCl, 1 CaCl_2_, 1 MgCl_2_, and 5 HEPES; pH 7.5.

### Subcloning, mutagenesis and in vitro transcription of nAChR subunits

Wild type nAChR subunits (hα6, hβ2 and hβ3) and gain-of-function nAChR hβ3 subunits (i.e., hβ3^V9’S^, where V9’S indicated valine-to-serine mutation in the so called 9’ position of the channel lining second transmembrane domain) were subcloned previously [26-28]. A synthetic nAChR hβ2 subunit with codon sequences optimized (GenBank Accession Number JN565027) for better heterologous expression was made (Invitrogen/GENEART, Burlingame, CA) and subcloned into the pCI vector (Promega, San Luis Obispo, CA) [26,28,38]. Missense mutations (i.e., Ser43Pro, Asn46lys, Asp57Asn, Arg87Cys, Asp92Glu, Arg96His, Glu101Lys, Ala112Val, Ser156Arg, Asn171Lys, Ala184Asp, Asp199Tyr, Asn203Thr, Ile226Thr and Ser233Cys) in the nAChR hα6 subunit were introduced in the pGEMHE (oocyte expression vector) background using HPLC purified oligonucleotides listed in Table 2. Accuracy of all mutant subunits were confirmed by sequencing referenced to nucleotide/protein sequences available in GenBank. Full length capped mRNA (i.e., cRNA) was transcribed from linearized plasmids using mMESSAGE mMACHINE® T7 Kit (Invitrogen/Ambion Inc., Carlsbad, CA). Integrity and quality of the cRNA was checked by electrophoresis and UV-spectroscopy.

### Preparation of cRNA mixture for Xenopus oocyte microinjection

We planned to introduce identical amounts of cRNA, presumably producing equal amounts of each subunit protein, into oocytes largely due to lack of information about the levels of mRNA for each subunits that compose α6*-nAChRs in neurons or other cells. Concentrations of cRNAs for each nAChR α and β subunits (WT, mutant or variant) were adjusted to 500 ng μL^−1^. We provisionally assumed that hα6 or its variants in association with hβ2 or hβ4 subunits would form complexes having 2:3 and/or 3:2 ratios of the indicated subunits and that oocytes also injected with WT or mutant form of β3 subunits (i.e., β3^V9’S^) would express nAChR with 2:2:1 ratios of α:β:(β3 or β3^V9’S^) subunits. For expression of binary nAChRs (i.e., two subunit containing nAChRs; α + β but not β3) cRNA mixtures were prepared by mixing 1 μL of cRNA for each subunit and an additional μL of RNAse free water (i.e., total volume 3 μL). Similarly for expression of ternary nAChRs [i.e., three subunit containing nAChRs; (α + β) + (β3 or β3^V9’S^)] cRNA mixtures were prepared by mixing 1 μL of cRNA for each subunit. Several preparations of each mixture were prepared and stored at −80°C until further use. Injection of 138 nL of cRNA, out of 3 μL cRNA mixtures, into each oocyte would deliver ~23 ng of cRNA for each subunit whether binary or ternary nAChRs are expressed.

### Oocyte preparation and cRNA microinjection

All *Xenopus laevis* (Nasco, Fort Atkinson, WI, USA) procedures were conducted in accordance with the guidelines of the National Institutes of Health (NIH) for the proper use of laboratory animals and approved by the Institutional Animal Care and Use Committee (IACU) of University of Virginia. Female *Xenopus laevis* (Nasco, Fort Atkinson, WI) were anesthetized using 0.2% tricaine methanesulfonate (MS-222) (Nasco, Fort Atkinson, WI). Ovarian lobes were surgically removed from the frogs and placed in an incubation solution that consisted of (in mM) 82.5 NaCl, 2.5 KCl, 1 MgCl2, 1 CaCl2, 1 Na_2_HPO_4_, 0.6 theophylline, 2.5 sodium pyruvate, 5 HEPES, 50 mg/ml gentamycin, 50 U/ml penicillin, and 50 μg/ml streptomycin; pH 7.5. The lobes were cut into small pieces and digested with Liberase™ (research grade, medium Thermolysin concentration; Roche Applied Science, Indianapolis, IN) with constant stirring at room temperature for 1.5-2 hours. The dispersed oocytes were thoroughly rinsed with incubation solution. Stage VI oocytes were selected and incubated at 16°C before injection. Micropipettes used for injection were pulled from borosilicate glass (Drummond Scientific, Broomall, PA) using a Sutter P1000 horizontal puller, and the tips were broken with forceps to ~40 μm in diameter. cRNA was drawn up into the micropipette and injected into oocytes using a Nanoject microinjection system (Drummond Scientific) at a total volume of 138 nL.

### Oocyte electrophysiology

Two to 5 days after injection, oocytes were placed in a small-volume chamber and continuously perfused with OR2. The chamber was grounded through an agarose bridge. The oocytes were voltage-clamped at −70 mV (unless otherwise noted) to measure agonist or antagonist induced currents using Axoclamp 900A and pClamp 10.2 software (Molecular Devices, Sunnyvale, CA). The current signal was low-pass filtered at 10 Hz with the built-in low-pass Bessel filter in the Axoclamp 900A and digitized at 20 Hz with Axon Digidata1440A and pClamp10. Electrodes contained 3 M KCl and had a resistance of 1–2 MΩ. Drugs (agonists and antagonists) were prepared daily in OR2. Drug was applied using a Valvelink 8.2 perfusion system (Automate scientific, Berkeley, CA). One micromolar (1 μM) atropine was always co-applied for acetylcholine (ACh)-based recordings to eliminate muscarinic AChR (mAChR) responses. nAChR hα6 constructs were tested individually or in batches as they became available to get an approximate idea about their effect on the function of hα6*-nAChRs. Then for the purpose of comparison electrophysiological recordings were performed in a given day in a given batch of oocytes following the same order of injections. Hence data points in a figure panel were obtained under almost similar experimental conditions. All hβ4*-nAChR recordings were done in similar conditions to facilitate comparisons between hα6hβ4- and hα6hβ4hβ3-nAChRs. All electrophysiological measurements were conducted or checked in at least two batches of oocytes.

### Experimental controls

Injection of water or empty vector or of cRNA corresponding to one subunit alone or pairwise combinations of β3 or β3^V9’S^ subunits with either an α or β2 or β4 subunit did not result in the expression of functional nAChR. Current responses from these oocytes to 100 μM nicotine were less than 5–10 nA (data not shown).

### Data analyses

Raw data was collected and processed in part using pClamp 10.2 (Molecular Devices, Sunnyvale, CA), Origin 7.5 (OriginLab Corporation, Northampton, MA) and a spreadsheet (Excel; Microsoft, Bellevue, WA). Peak current amplitudes (I_max_) are reported as mean ± SEM (for results from at least three oocytes, i.e., n = 3). Concentration-response (CR) relationships, in which mean peak current amplitudes at specified ligand concentrations were fit to the Hill equation or its variants using Prism 4 (GraphPad Software, San Diego, CA), were constructed to assess true I_max_ (mean current amplitudes in response to the most efficacious concentration of an agonist) and EC_50_ (concentration for half-maximal activation) values. The F-test (p < 0.05 to define statistical significance) was used to compare the best-fit values of log molar EC_50_ values across specific nAChR subunit combinations. EC_50_ values with non-overlapping 95% confidence intervals (CI) are deemed to be statistically significant (p < 0.05).

There are limitations in the ability to compare peak current responses of nAChRs, even though we injected similar amounts of cRNAs for 1:1 or 1:1:1 coexpressions, as described previously [27,28]. This is because expression levels assessed as peak current amplitudes are affected by batch-to-batch variation in oocytes, time between cRNA injection and recording, and subunit combination-specific parameters, such as open probability (influenced by gating rate constants, rates and extents of desensitization), single channel conductance, assembly efficiency, and efficiency of receptor trafficking to the cell surface [46]. We made no attempt to measure or control for subunit combination-specific effects, but whenever preliminary studies revealed possible differences in peak current amplitudes, findings were further confirmed across different subunit combinations using the same batch of oocytes and the same time between cRNA injection and recording. However, when we make statements about results comparing ligand potencies and peak current amplitudes across subunit combinations, we do so for studies done under the same or very similar conditions, and the observations are clear, statistically significant, and in agreement whether for pooled data or for results from smaller sets of studies. The I_max_ values of the WT and variant hα6*-nAChRs were compared using Student’s t-test (two-tailed. *, p < 0.05; **, p < 0.01; and ***, p < 0.001) or ANOVA (followed by Tukey’s or Dunnett’s multiple comparisons test; *, p < 0.05; **, p < 0.01; and ***, p < 0.001).

## Abbreviations

ACh: Acetylcholine; nAChR: Nicotinic acetylcholine receptor(s); Imax: Peak current response; SNP: Single nucleotide polymorphism; SNV: Single nucleotide variation; CR: Concentration-response; h: Human; AA: Amino acid.

## Competing interests

The authors do not have any conflicting or competing interests.

## Authors’ contributions

BD and MDL conceived the project. BD designed and conducted all the experiments, analyzed the data and drafted the manuscript. MDL wrote or contributed to the writing of the manuscript. Both authors have read and approved the final submitted version of this manuscript.

## Supplementary Material

Additional file 1: Figure S1 Variations in nAChR hα6 subunit influence the nicotine sensitivity of hα6hβ4*-nAChRs. **Table S1.** Parameters for nicotine action at WT or variant hα6hβ4*- nAChRs. Click here for file
