# Supplementary material for: Analysis of rare variations reveals roles of amino acid residues in the N-terminal extracellular domain of nicotinic acetylcholine receptor (nAChR) alpha6 subunit in the functional expression of human alpha6*-nAChRs
Source: Mol Brain. 2014 May 2;7:35. doi: 10.1186/1756-6606-7-35 (PMC4022547; doi:10.1186/1756-6606-7-35)
Supplement: Additional file 1: Figure S1 — Variations in nAChR hα6 subunit influence the nicotine sensitivity of hα6hβ4*-nAChRs. Table S1. Parameters for nicotine action at WT or variant hα6hβ4*- nAChRs. [file 1756-6606-7-35-S1.docx]

**Analysis of rare variations reveals roles of amino acid residues in the N-terminal extracellular domain of nicotinic acetylcholine receptor (nAChR) alpha6 subunit in the functional expression of human alpha6*-nAChRs**

Bhagirathi Dash and Ming D. Li

Department of Psychiatry and Neurobehavioral Sciences, School of Medicine, University of Virginia, Charlottesville, VA

**Supplementary information**


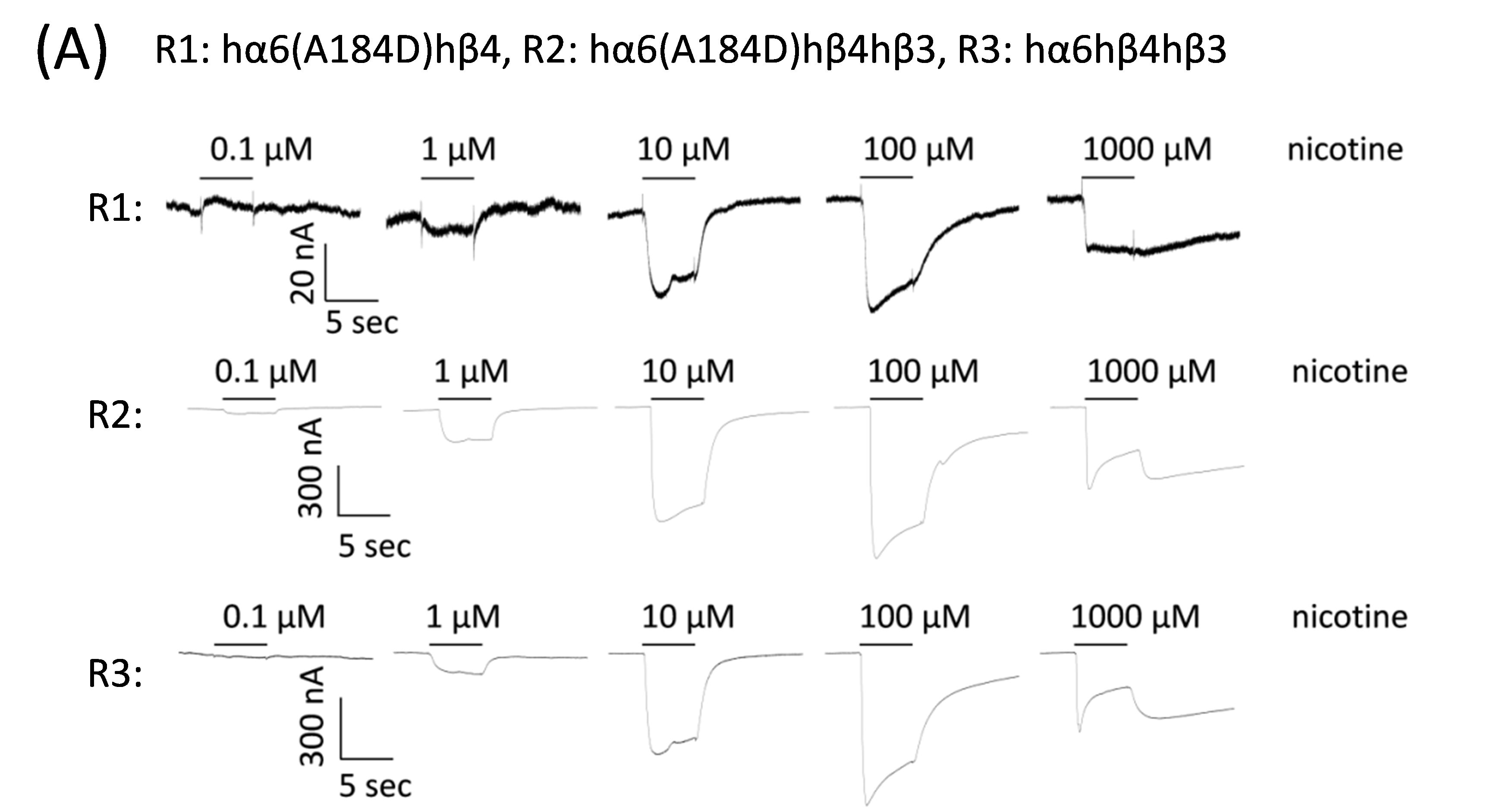


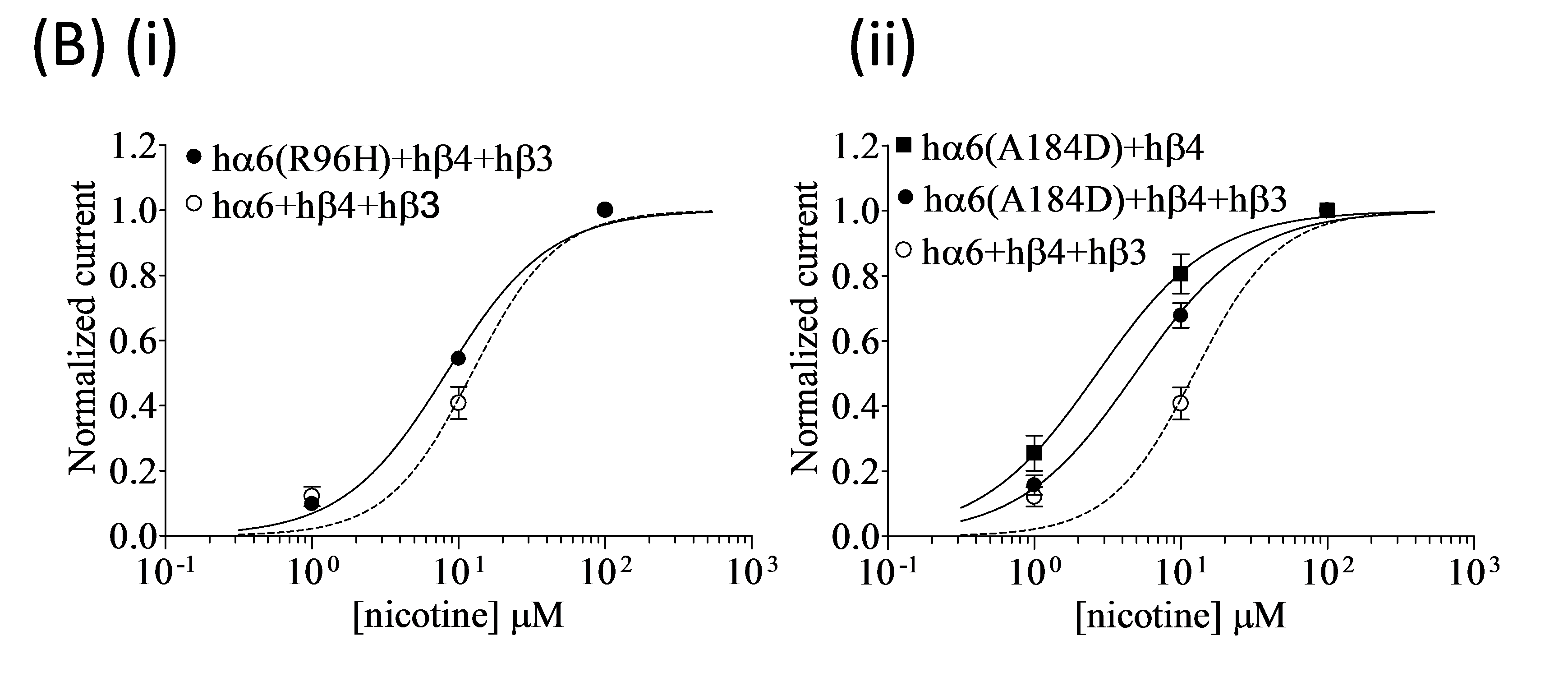


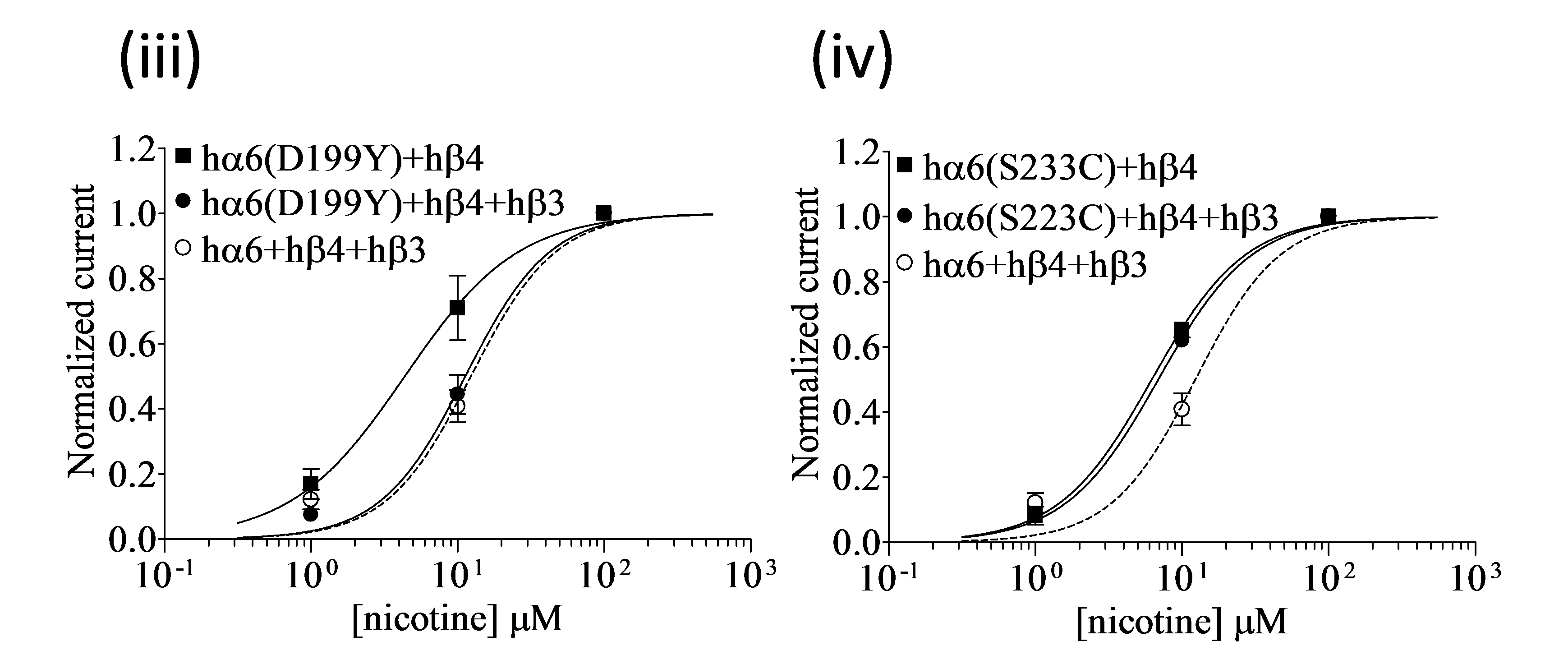


**Figure S1. Variations in nAChR hα6 subunit influence the nicotine sensitivity of hα6hβ4*-nAChRs.** (**A**) Representative traces are shown for current responses from oocytes (voltage clamped at -70 mV) responding to the application of indicated concentrations of nicotine (shown with the duration of drug exposure as black bars above the traces) and expressing indicated nAChR (i.e., R1: hα6^A184D^hβ4-nAChR, R2: hα6^A184D^hβ4hβ3-nAChR, R3: hα6hβ4hβ3-nAChR). (**B**) Results averaged across experiments were used to produce concentration-response (CR) curves (ordinate-mean normalized current ± SEM; abscissa - ligand concentration in log μM) for inward current responses to nicotine as indicated for the nAChR expressed in oocytes and voltage clamped at -70 mV. Current amplitudes are represented as a fraction of the peak inward current amplitude in response to the most efficacious concentration of nicotine. Leftward shifts in nicotine CR curves for hα6^R96H^hβ4hβ3-(●) [**(B) (i)**], hα6^A184D^hβ4hβ3-(●) [**(B) (ii)**], hα6^D199Y^hβ4hβ3-(●) [**(B) (iii)**], or hα6^S233C^hβ4hβ3-(●) [**(B) (iv)**] nAChR are evident relative to that of hα6hβ4hβ3-nAChR (○). Furthermore nicotine curves for hα6^A184D^hβ4-(◼) [**(B) (ii)**], hα6^D199Y^hβ4-(◼) [**(B) (iii)**], or hα6^S233C^hβ4-(◼) [**(B) (iv)**] nAChR are shifted leftward relative to those nAChR containing the same subunits but in the additional presence of hβ3 subunits. See Table S1 for parameters of nicotine action.

**Table S1. Parameters for nicotine action at WT or variant hα6hβ4*- nAChRs**

Potencies [micromolar EC_50_ values with 95% confidence intervals (CI)], Hill coefficients (n_H_ ± SE) and concentrations (μM) where maximal peak current amplitudes (I_max_) achieved are provided for nicotine acting at nAChR composed of the indicated subunits and from the indicated number of independent experiments (n) based on studies as shown in Fig S1. 🡹 or 🡻 indicate a significant (p<0.05) increase or decrease in indicated parameter at the indicated nAChR subtype relative to nAChR containing the same subunits but in the presence of the WT hα6 subunit (i.e., hα6hβ4- vs. variant-hα6hβ4- nAChR; hα6hβ4hβ3- vs. variant-hα6hβ4hβ3- nAChR). ▲ or ▼ indicate a significant (p<0.05) increase or decrease, respectively, in indicated parameter at the indicated nAChR subtype relative to nAChR containing the same subunits but in the absence WT β3 subunits (i.e., hα6hβ4- vs. hα6hβ4hβ3- nAChR; varinat-hα6hβ4- vs. variant-hα6hβ4hβ3- nAChR). ‘-‘ indicates that inconsistent functional responses in two electrode voltage clamp studies precluded determination of the parameter of interest.

| nAChR subunit  combinations | n | EC_50_ (μM)  (95 % CI) | n_H_ ± SE n_H_ | I_max_ conc.  (µM) |
| --- | --- | --- | --- | --- |
| hα6+hβ4 |  | 7.1^1^ | - | 100 |
| hα6(R96H)+hβ4 | 3 | - | - | 100 |
| hα6(A184D)+hβ4 | 3 | 2.7 (1.8-3.9) | 1.1±0.15 | 100 |
| hα6(D199Y)+hβ4 | 3 | 4.4 (2.4-8) | 1.1±0.26 | 100 |
| hα6(S233C)+hβ4 | 3 | 6.2 (5.2-7.4) | 1.4±0.13 | 100 |
|  |  |  |  |  |
| hα6+hβ4+hβ3 | 4 | 12 (9.2-17) | 1.5±0.45 | 100 |
| hα6(R96H)+hβ4+hβ3 | 3 | 8.2 (6.9-9.8) | 1.2±0.13 | 100 |
| hα6(A184D)+hβ4+hβ3 | 3 | 4.9 (3.8-6.3) 🡻 | 1.1±0.11 | 100 |
| hα6(D199Y)+hβ4+hβ3 | 3 | 11 (8.7-15) ▲ | 1.5±0.42 | 100 |
| hα6(S233C)+hβ4+hβ3 | 3 | 6.9 (6-7.9) 🡻 | 1.4±0.12 | 100 |

^1^ From Kuryatov et al (2000)
